# Crystal structure of metronidazolium tetra­chlorido­aurate(III)

**DOI:** 10.1107/S2056989015010798

**Published:** 2015-06-17

**Authors:** Patrick J. Quinlivan, Ja-Shin Wu, Rita K. Upmacis

**Affiliations:** a Department of Chemistry, Columbia University, New York, NY 10027, USA; b Haskins Laboratories, Dept. of Chemistry, Pace University, New York, NY 10038, USA

**Keywords:** crystal structure, gold, metronidazole, flag­yl, tetra­chlorido­aurate(III), O—H⋯Cl hydrogen bonding

## Abstract

Metronidazole (MET) [systematic names: 1-(2-hy­droxy­eth­yl)-2-methyl-5-nitro-1*H*-imidazole and 2-(2-methyl-5-nitro-1*H*-imidazol-1-yl)ethanol] is a medication that is used to treat infections from a variety of anaerobic organisms. As with other imidazole derivatives, metronidazole is also susceptible to protonation. However, there are few reports of the structures of metronidazolium derivatives. In the title compound, (C_6_H_10_N_3_O_3_)[AuCl_4_] [systematic name: 1-(2-hy­droxy­eth­yl)-2-methyl-5-nitro-1*H*-imidazol-3-ium tetra­chlorido­aur­ate(III)], the asymmetric unit consists of a metronidazolium cation, [H(MET)]^+^, and a tetra­chlorido­aurate(III) anion, [AuCl_4_]^−^, in which the Au^III^ ion is in a slightly distorted square-planar coordination environment. In the cation, the nitro group is essentially coplanar with the imidazole ring, as indicated by an O N—C=C torsion angle of −0.2 (4)°, while the hy­droxy­ethyl group is in a coiled conformation, with an O(H)—C—C—N torsion angle of 62.3 (3)°. In the crystal, the anion and cation are linked by an inter­molecular O—H⋯Cl hydrogen bond. In addition, the N—H group of the metronidazolium ion serves as a hydrogen-bond donor to the O atom of the hy­droxy­ethyl group of a symmetry-related mol­ecule, leading to the formation of chains along [010].

## Abstract 2

Metronidazole (MET) reacts with HAuCl_4_·H_2_O to give metronidazolium tetra­chlorido­aurate(III), [H(MET)][AuCl_4_], in which the cation and anion are linked *via* an O—H⋯Cl hydrogen-bonding inter­action.

## Chemical context   

Metronidazole (MET), marketed as flagyl, and also known by the systematic names 1-(2-hy­droxy­eth­yl)-2-methyl-5-nitro-1*H*-imidazole and 2-(2-methyl-5-nitro-1*H*-imidazol-1-yl)ethanol, is a medication that has been used for the treatment of parasitic infections, such as trichomoniasis, amoebiasis and giardiasis, and is also effective against anaerobic bacteria (Freeman *et al.*, 1997 ▸; Miljkovic *et al.*, 2014 ▸; Soares *et al.*, 2012 ▸; Samuelson, 1999 ▸; Lofmark *et al.*, 2010 ▸; Contreras *et al.*, 2009 ▸). Metronidazole possesses a variety of functional groups, and the two-coordinate nitro­gen atom of the imidazole ring has been shown to be an effective ligand for a variety of metals (Contreras *et al.*, 2009 ▸). This nitro­gen atom is also susceptible to protonation, but there are few structures of metronidazolium derivatives reported in the literature (Yang, 2008 ▸; Wang *et al.*, 2010 ▸). We describe herein the structure of metronidazolium tetra­chlorido­aurate(III), which is obtained by the addition of MET to HAuCl_4_.

## Structural commentary   

**Figure 1:**
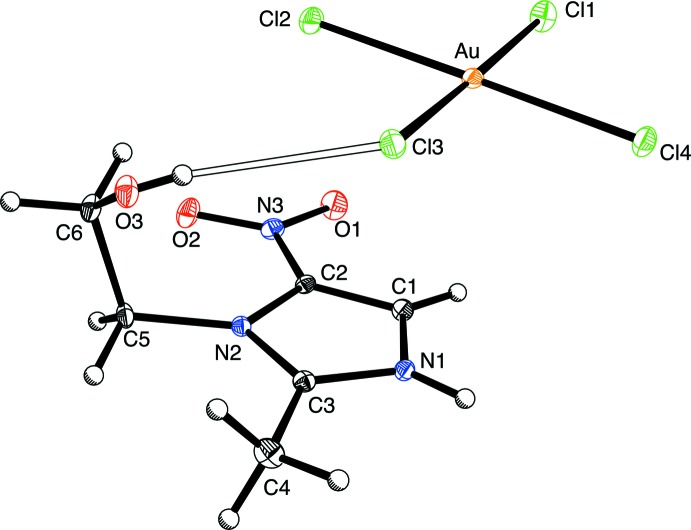
The asymmetric unit of the title compound, shown with 20% probability displacement ellipsoids. The O3—H3⋯Cl3 hydrogen bond is shown as an open bond.

The asymmetric unit of [H(MET)][AuCl_4_] consists of a metronidazolium cation, [H(MET)]^+^, hydrogen-bonded to a square-planar tetra­chlorido­aurate(III) anion, [AuCl_4_]^−^, by an O—H⋯Cl hydrogen bond as illustrated in Fig. 1 ▸. The O3⋯Cl3 distance of 3.169 (2) Å is comparable to the values in other tetra­chlorido­aurate(III) derivatives that exhibit O—H⋯Cl hydrogen bonds. As an illustration, bis­{2-[(2-hy­droxy­eth­yl)imino­meth­yl]phenolato}gold(III) tetra­chlorido­aurate(III) possesses an O—H⋯Cl hydrogen bond between a hy­droxy­ethyl group and [AuCl_4_]^−^, with an O(H)⋯Cl distance of 3.365 Å (Nockemann *et al.*, 2007 ▸). For further reference, the average O⋯Cl distance in compounds that have O—H⋯Cl inter­actions is 3.196 (3) Å (Steiner, 2002 ▸). The nitro group is almost coplanar with the imidazole ring, as indicated by an O1—N3—C2—C1 torsion angle of −0.2 (4)°, while the hy­droxy­ethyl group exhibits an O3—C6—C5—N2 torsion angle of 62.3 (3)°, describing a coiled conformation.
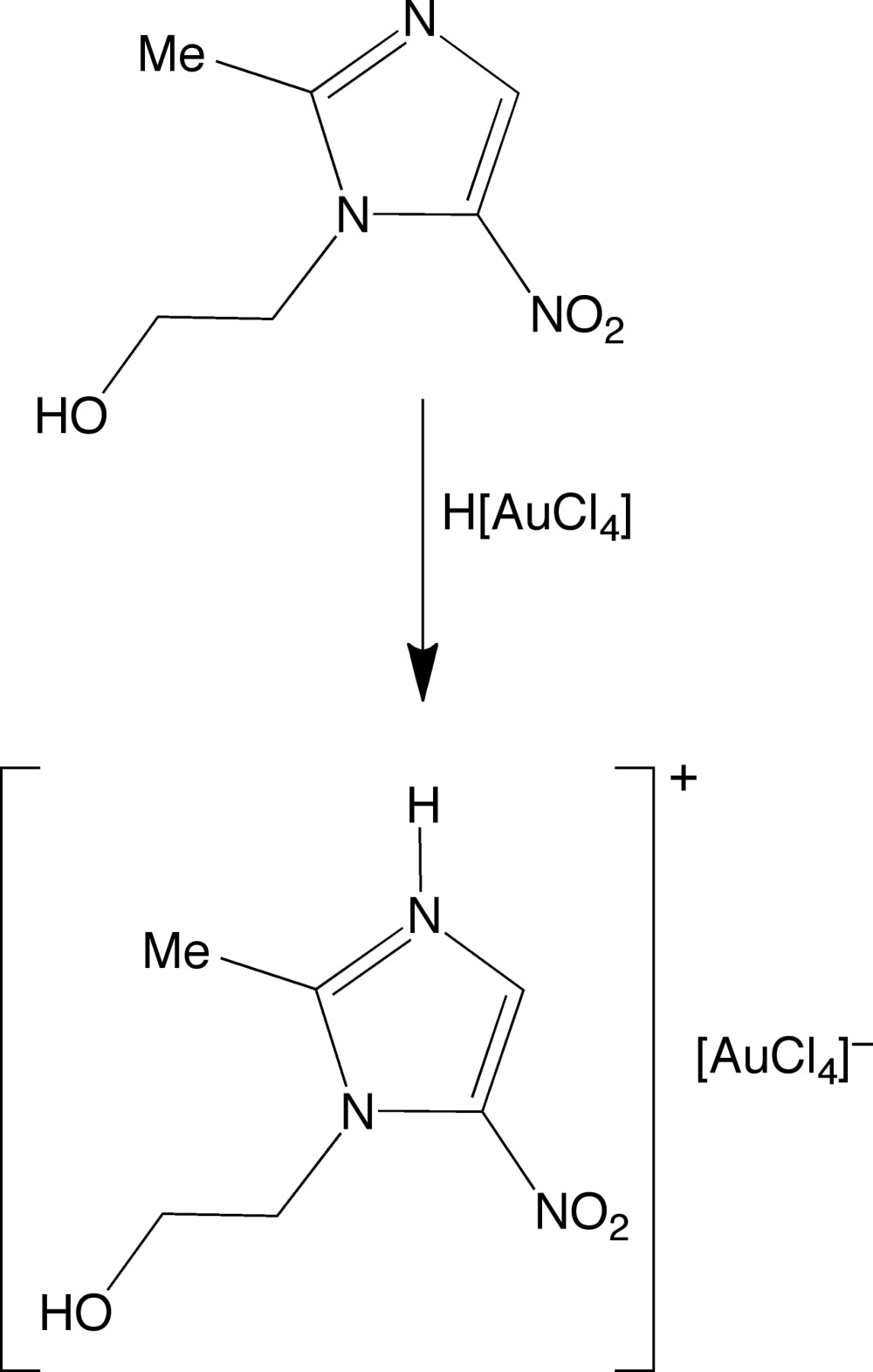


## Supra­molecular features   

**Figure 2:**
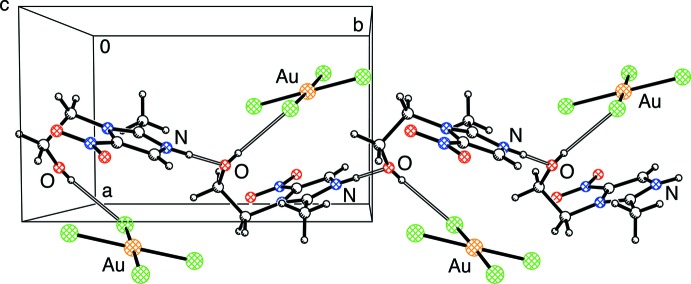
Part of the crystal structure showing a hydrogen-bonded chain (open bonds) along [010].

In the crystal, the N—H group of the metronidazolium ion serves as a hydrogen-bond donor to the oxygen atom of the hy­droxy­ethyl group of a symmetry-related mol­ecule, forming a chain along [010] in which each O—H group is O—H⋯Cl hydrogen bonded to a [AuCl_4_]^−^ ion (Table 1 ▸ and Fig. 2 ▸). The N⋯O distance of 2.729 (3) Å associated with the hydrogen bond is comparable to that observed for metronidazole [2.816 (2) Å] (Blaton *et al.*, 1979 ▸; Galván-Tejada *et al.*, 2002 ▸). However, an important difference between the hydrogen bonds in metronidazole and metronidazolium is that the alcohol O—H group is the hydrogen-bond donor for metronidazole (*i.e.* O—H⋯N), while the N—H group is the hydrogen-bond donor for metronidazolium (*i.e.* N—H⋯O).

## Database survey   

Metronidazolium derivatives that feature other counter-ions, *e.g.* 3-carb­oxy-4-hy­droxy­benzene­sulfonate and perchlorate have been reported (Yang, 2008 ▸; Wang *et al.*, 2010 ▸), as have a variety of tetra­chlorido­aurate(III) complexes (Johnson & Steed, 1998 ▸; Pluzhnik-Gladyr *et al.*, 2014 ▸; Faza­eli *et al.*, 2010 ▸).

## Synthesis and crystallization   

Crystals of composition [H(MET)][AuCl_4_] were obtained by combining HAuCl_4_·H_2_O (0.12 mmol) with MET (0.20 mmol) in MeOH (2 ml), followed by evaporation of MeOH, and crystallization from Et_2_O.

## Refinement   

Crystal data, data collection and structure refinement details are summarized in Table 2 ▸. H atoms bonded to C atoms were refined with a riding model, with C—H = 0.95–0.99 Å and *U*_iso_(H) = 1.2*U*_eq_(C) or 1.5*U*_eq_(C_meth­yl_). H atoms bonded to N and O atoms were refined independently with isotropic displacement parameters.

## Supplementary Material

Crystal structure: contains datablock(s) I. DOI: 10.1107/S2056989015010798/lh5766sup1.cif

Structure factors: contains datablock(s) I. DOI: 10.1107/S2056989015010798/lh5766Isup3.hkl

CCDC reference: 1404845

Additional supporting information:  crystallographic information; 3D view; checkCIF report

## Figures and Tables

**Table 1:** Hydrogen-bond geometry (, )

*D*H*A*	*D*H	H*A*	*D* *A*	*D*H*A*
N1H01O3^i^	0.94(4)	1.81(4)	2.729(3)	166(3)
O3H3Cl3	0.67(4)	2.54(4)	3.169(2)	158(4)

Symmetry code: (i) 
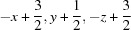
.

**Table 2:** Experimental details

Crystal data	
Chemical formula	(C_6_H_10_N_3_O_3_)[AuCl_4_]
*M*_r_	510.94
Crystal system, space group	Monoclinic, *P*2_1_/*n*
Temperature (K)	130
*a*, *b*, *c* ()	7.324(2), 11.972(4), 15.667(5)
()	94.384(4)
*V* (^3^)	1369.6(8)
*Z*	4
Radiation type	Mo *K*
(mm^1^)	11.52
Crystal size (mm)	0.23 0.04 0.02

Data collection	
Diffractometer	Bruker APEXII CCD
Absorption correction	Multi-scan (*SADABS*; Bruker, 2013 ▸)
*T*_min_, *T*_max_	0.426, 0.746
No. of measured, independent and observed [*I* > 2(*I*)] reflections	22024, 4214, 3673
*R*_int_	0.041
(sin /)_max_ (^1^)	0.718

Refinement	
*R*[*F* ^2^ > 2(*F* ^2^)], *wR*(*F* ^2^), *S*	0.021, 0.045, 1.16
No. of reflections	4214
No. of parameters	163
H-atom treatment	H atoms treated by a mixture of independent and constrained refinement
_max_, _min_ (e ^3^)	1.30, 1.22

Computer programs: *APEX2* and, *SAINT* (Bruker, 2013 ▸), *SHELXS97* and *SHELXTL* (Sheldrick, 2008 ▸), *SHELXL2014* (Sheldrick, 2015 ▸).

## supplementary crystallographic information

### Crystal data

**Table:**

(C_6_H_10_N_3_O_3_)[AuCl_4_]	*F*(000) = 952
*M**_r_* = 510.94	*D*_x_ = 2.478 Mg m^−^^3^
Monoclinic, *P*2_1_/*n*	Mo *K*α radiation, λ = 0.71073 Å
*a* = 7.324 (2) Å	Cell parameters from 9874 reflections
*b* = 11.972 (4) Å	θ = 2.6–30.6°
*c* = 15.667 (5) Å	µ = 11.52 mm^−^^1^
β = 94.384 (4)°	*T* = 130 K
*V* = 1369.6 (8) Å^3^	Plate, yellow
*Z* = 4	0.23 × 0.04 × 0.02 mm

### Data collection

**Table:**

Bruker APEXII CCD diffractometer	3673 reflections with *I* > 2σ(*I*)
φ and ω scans	*R*_int_ = 0.041
Absorption correction: multi-scan (*SADABS*; Bruker, 2013)	θ_max_ = 30.7°, θ_min_ = 2.1°
*T*_min_ = 0.426, *T*_max_ = 0.746	*h* = −10→10
22024 measured reflections	*k* = −17→17
4214 independent reflections	*l* = −22→22

### Refinement

**Table:**

Refinement on *F*^2^	0 restraints
Least-squares matrix: full	Hydrogen site location: mixed
*R*[*F*^2^ > 2σ(*F*^2^)] = 0.021	H atoms treated by a mixture of independent and constrained refinement
*wR*(*F*^2^) = 0.045	*w* = 1/[σ^2^(*F*_o_^2^) + (0.0129*P*)^2^] where *P* = (*F*_o_^2^ + 2*F*_c_^2^)/3
*S* = 1.16	(Δ/σ)_max_ = 0.001
4214 reflections	Δρ_max_ = 1.30 e Å^−^^3^
163 parameters	Δρ_min_ = −1.22 e Å^−^^3^

### Special details

**Table:**

Geometry. All e.s.d.'s (except the e.s.d. in the dihedral angle between two l.s. planes) are estimated using the full covariance matrix. The cell e.s.d.'s are taken into account individually in the estimation of e.s.d.'s in distances, angles and torsion angles; correlations between e.s.d.'s in cell parameters are only used when they are defined by crystal symmetry. An approximate (isotropic) treatment of cell e.s.d.'s is used for estimating e.s.d.'s involving l.s. planes.

### Fractional atomic coordinates and isotropic or equivalent isotropic displacement parameters (Å^2^)

**Table:**

	*x*	*y*	*z*	*U*_iso_*/*U*_eq_	
Au	0.36629 (2)	0.79983 (2)	0.58625 (2)	0.01868 (4)	
Cl1	0.26070 (11)	0.84595 (7)	0.45047 (5)	0.03247 (17)	
Cl2	0.44092 (10)	0.62541 (6)	0.54111 (5)	0.03012 (16)	
Cl3	0.46029 (10)	0.75210 (6)	0.72389 (5)	0.02610 (15)	
Cl4	0.29498 (10)	0.97418 (6)	0.63205 (5)	0.02831 (15)	
O1	0.8047 (3)	0.7553 (2)	0.41611 (14)	0.0367 (5)	
O2	0.9242 (3)	0.60835 (18)	0.47815 (14)	0.0331 (5)	
O3	0.7450 (3)	0.55236 (18)	0.73360 (15)	0.0265 (5)	
H3	0.684 (5)	0.591 (3)	0.719 (2)	0.042 (13)*	
N1	0.8446 (3)	0.89061 (19)	0.65413 (15)	0.0175 (4)	
H01	0.810 (5)	0.953 (3)	0.685 (2)	0.042 (10)*	
N2	0.9387 (3)	0.72022 (17)	0.63739 (14)	0.0159 (4)	
N3	0.8670 (3)	0.70319 (19)	0.47846 (15)	0.0223 (5)	
C1	0.8127 (4)	0.8685 (2)	0.56913 (17)	0.0191 (5)	
H1A	0.7604	0.9173	0.5261	0.023*	
C2	0.8708 (3)	0.7626 (2)	0.55853 (17)	0.0158 (5)	
C3	0.9200 (4)	0.8016 (2)	0.69521 (17)	0.0170 (5)	
C4	0.9783 (4)	0.7982 (3)	0.78720 (19)	0.0272 (6)	
H4A	0.9318	0.8643	0.8154	0.041*	
H4B	1.1123	0.7972	0.7949	0.041*	
H4C	0.9295	0.7307	0.8126	0.041*	
C5	1.0107 (4)	0.6070 (2)	0.65912 (18)	0.0198 (5)	
H5A	1.0863	0.6102	0.7142	0.024*	
H5B	1.0904	0.5822	0.6145	0.024*	
C6	0.8579 (4)	0.5230 (2)	0.66604 (18)	0.0227 (6)	
H6A	0.7818	0.5199	0.6111	0.027*	
H6B	0.9111	0.4479	0.6773	0.027*	

### Atomic displacement parameters (Å^2^)

**Table:**

	*U*^11^	*U*^22^	*U*^33^	*U*^12^	*U*^13^	*U*^23^
Au	0.01490 (5)	0.01746 (6)	0.02409 (6)	−0.00147 (4)	0.00416 (4)	−0.00253 (4)
Cl1	0.0438 (4)	0.0304 (4)	0.0234 (4)	0.0042 (3)	0.0037 (3)	−0.0009 (3)
Cl2	0.0286 (4)	0.0222 (3)	0.0400 (4)	0.0024 (3)	0.0057 (3)	−0.0092 (3)
Cl3	0.0255 (3)	0.0251 (3)	0.0272 (4)	0.0036 (3)	−0.0008 (3)	−0.0012 (3)
Cl4	0.0360 (4)	0.0193 (3)	0.0292 (4)	0.0033 (3)	0.0002 (3)	−0.0038 (3)
O1	0.0516 (15)	0.0375 (13)	0.0193 (11)	0.0047 (11)	−0.0090 (10)	−0.0008 (10)
O2	0.0468 (14)	0.0221 (11)	0.0309 (12)	0.0056 (10)	0.0058 (10)	−0.0077 (9)
O3	0.0250 (11)	0.0233 (11)	0.0327 (13)	0.0053 (9)	0.0109 (9)	0.0075 (9)
N1	0.0172 (10)	0.0152 (10)	0.0203 (11)	−0.0003 (9)	0.0023 (9)	−0.0003 (9)
N2	0.0146 (10)	0.0157 (10)	0.0175 (11)	0.0000 (8)	0.0021 (8)	0.0014 (8)
N3	0.0253 (12)	0.0233 (12)	0.0181 (12)	−0.0032 (10)	0.0010 (9)	−0.0019 (9)
C1	0.0217 (13)	0.0198 (13)	0.0157 (12)	0.0003 (10)	−0.0001 (10)	0.0012 (10)
C2	0.0184 (12)	0.0152 (11)	0.0138 (12)	−0.0023 (10)	0.0008 (9)	−0.0006 (9)
C3	0.0146 (12)	0.0188 (12)	0.0180 (13)	−0.0012 (10)	0.0038 (10)	0.0002 (10)
C4	0.0292 (16)	0.0355 (17)	0.0167 (14)	0.0031 (13)	0.0011 (12)	−0.0009 (12)
C5	0.0181 (12)	0.0170 (12)	0.0245 (14)	0.0051 (10)	0.0026 (10)	0.0064 (10)
C6	0.0254 (14)	0.0164 (13)	0.0271 (15)	0.0029 (11)	0.0072 (12)	0.0046 (11)

### Geometric parameters (Å, º)

**Table:**

Au—Cl1	2.2752 (10)	N2—C5	1.485 (3)
Au—Cl4	2.2807 (9)	N3—C2	1.441 (3)
Au—Cl2	2.2844 (9)	C1—C2	1.351 (4)
Au—Cl3	2.2855 (10)	C1—H1A	0.9500
O1—N3	1.218 (3)	C3—C4	1.472 (4)
O2—N3	1.210 (3)	C4—H4A	0.9800
O3—C6	1.436 (3)	C4—H4B	0.9800
O3—H3	0.67 (4)	C4—H4C	0.9800
N1—C3	1.341 (3)	C5—C6	1.515 (4)
N1—C1	1.360 (3)	C5—H5A	0.9900
N1—H01	0.94 (4)	C5—H5B	0.9900
N2—C3	1.345 (3)	C6—H6A	0.9900
N2—C2	1.392 (3)	C6—H6B	0.9900
			
Cl1—Au—Cl4	90.14 (3)	N1—C3—N2	108.2 (2)
Cl1—Au—Cl2	90.27 (3)	N1—C3—C4	124.7 (2)
Cl4—Au—Cl2	179.36 (3)	N2—C3—C4	127.0 (2)
Cl1—Au—Cl3	177.66 (3)	C3—C4—H4A	109.5
Cl4—Au—Cl3	89.52 (3)	C3—C4—H4B	109.5
Cl2—Au—Cl3	90.09 (3)	H4A—C4—H4B	109.5
C6—O3—H3	108 (3)	C3—C4—H4C	109.5
C3—N1—C1	110.4 (2)	H4A—C4—H4C	109.5
C3—N1—H01	120 (2)	H4B—C4—H4C	109.5
C1—N1—H01	129 (2)	N2—C5—C6	111.8 (2)
C3—N2—C2	106.6 (2)	N2—C5—H5A	109.3
C3—N2—C5	124.1 (2)	C6—C5—H5A	109.3
C2—N2—C5	129.3 (2)	N2—C5—H5B	109.3
O2—N3—O1	125.9 (3)	C6—C5—H5B	109.3
O2—N3—C2	118.9 (2)	H5A—C5—H5B	107.9
O1—N3—C2	115.2 (2)	O3—C6—C5	111.1 (2)
C2—C1—N1	105.7 (2)	O3—C6—H6A	109.4
C2—C1—H1A	127.1	C5—C6—H6A	109.4
N1—C1—H1A	127.1	O3—C6—H6B	109.4
C1—C2—N2	109.1 (2)	C5—C6—H6B	109.4
C1—C2—N3	125.8 (2)	H6A—C6—H6B	108.0
N2—C2—N3	125.1 (2)		

### Hydrogen-bond geometry (Å, º)

**Table:**

*D*—H···*A*	*D*—H	H···*A*	*D*···*A*	*D*—H···*A*
N1—H01···O3^i^	0.94 (4)	1.81 (4)	2.729 (3)	166 (3)
O3—H3···Cl3	0.67 (4)	2.54 (4)	3.169 (2)	158 (4)

Symmetry code: (i) −*x*+3/2, *y*+1/2, −*z*+3/2.
